# Antimicrobial Activity of Ceragenins against Vancomycin-Susceptible and -Resistant *Enterococcus* spp.

**DOI:** 10.3390/ph16121643

**Published:** 2023-11-23

**Authors:** Mayram Hacioglu, Fatima Nur Yilmaz, Ozlem Oyardi, Cagla Bozkurt Guzel, Nese Inan, Paul B. Savage, Sibel Dosler

**Affiliations:** 1 Department of Pharmaceutical Microbiology, Faculty of Pharmacy, Istanbul University, Istanbul 34116, Turkey; f.yilmaz@istanbul.edu.tr (F.N.Y.); cagla.bozkurt@istanbul.edu.tr (C.B.G.); sibel.dosler@istanbul.edu.tr (S.D.); 2 Department of Pharmaceutical Microbiology, Faculty of Pharmacy, Gazi University, Ankara 06330, Turkey; ozlemoyardi@gazi.edu.tr; 3 Medical Microbiology Laboratory, Dr. Abdurrahman Yurtaslan Oncology Training and Research Hospital, University of Health Sciences Ankara, Ankara 06200, Turkey; neseurdogan@yahoo.com; 4 Department of Chemistry and Biochemistry, Brigham Young University, Provo, UT 84602, USA; pbsavage@chem.byu.edu

**Keywords:** *Enterococcus* spp., ceragenin, linezolid, minimum inhibitory concentration, microbroth checkerboard, *Caenorhabditis elegans*

## Abstract

Ceragenins (CSAs) are a new class of antimicrobial agents designed to mimic the activities of endogenous antimicrobial peptides. In this study, the antibacterial activities of various ceragenins (CSA-13, CSA-44, CSA-90, CSA-131, CSA-138, CSA-142, and CSA-192), linezolid, and daptomycin were assessed against 50 non-repeated *Enterococcus* spp. (17 of them vancomycin-resistant Enterococcus-VRE) isolated from various clinical specimens. Among the ceragenins evaluated, the MIC_50_ and MIC_90_ values of CSA-44 and CSA-192 were the lowest (2 and 4 μg/mL, respectively), and further studies were continued with these two ceragenins. Potential interactions between CSA-44 or CSA-192 and linezolid were tested and synergistic interactions were seen with the CSA-192-linezolid combination against three *Enterococcus* spp., one of them VRE. The effects of CSA-44 and CSA-192 on the MIC values of vancomycin were also investigated, and the largest MIC change was seen in the vancomycin-CSA-192 combination. The in vivo effects of CSA-44 and CSA-192 were evaluated in a *Caenorhabditis elegans* model system. Compared to no treatment, increased survival was observed with *C. elegans* when treated with ceragenins. In conclusion, CSA-44 and CSA-192 appear to be good candidates (alone or in combination) for the treatment of enterococcal infections, including those from VRE.

## 1. Introduction

*Enterococcus* spp. are Gram-positive bacteria that have been isolated from soil, surface waters, seawater, plants, and fermented food products. In addition to being the leading commensal members of the gastrointestinal tract, particularly the small and large intestines in humans and animals, they can be causative agents of human diseases. *Enterococcus* spp. are opportunistic pathogens capable of translocating across gastrointestinal membranes and causing systemic infections, especially in immunocompromised and hospitalized patients [1,2]. The dominant species within this genus are *E. faecalis* and *E. faecium*. They can generate common and difficult-to-treat infections due to their success in developing antimicrobial resistance and their biofilm-forming abilities in environments such as hospitals and patient care centers. Among these diseases, bacteremia, urinary tract infections, intra-abdominal infections, endocarditis, and wound infection can be listed as the main infections [2]. Enterococcal bacteremia is linked to a substantial mortality rate, ranging between 25% and 50%. The predominant etiology of enterococcal bacteremia is believed to stem from the translocation of *Enterococcus* spp. from the gastrointestinal tract into the bloodstream. Factors contributing to elevated mortality risk in enterococcal bacteremia include the patient’s age, the severity of the illness, and prolonged utilization of antibiotics [3]. One of the most common infections caused by *Enterococcus* spp. is urinary tract infections, which occur particularly in healthcare settings such as hospitals and long-term care facilities and are often multidrug-resistant. There is a risk of urinary tract infections turning into bacteremia in these patients. Approximately 15% of urinary tract infections diagnosed in intensive care patients are caused by *Enterococcus* spp. [1].

*Enterococcus* spp. are among the ESKAPE pathogens, which are major causes of hospital-acquired infections [4,5,6]. The Infectious Diseases Society of America (IDSA) has stated that urgent precautions should be taken against these organisms. ESKAPE is an acronym for *Enterococcus faecium*, *Staphylococcus aureus*, *Klebsiella pneumoniae*, *Acinetobacter baumannii*, *Pseudomonas aeruginosa*, and *Enterobacter* species. ESKAPE pathogens are microorganisms that have high antibiotic resistance and can spread rapidly in hospitals, healthcare settings, and long-term care facilities, and urgent actions are required to prevent their spread [7].

*Enterococcus* spp. show intrinsic resistance to many antimicrobials as well as acquired resistance. *Enterococcus* spp. have intrinsic resistance to antimicrobials such as nearly all cephalosporins, antistaphylococcal penicillins, aztreonam, clindamycin, and trimethoprim-sulfamethoxazole. Although there is no intrinsic resistance to tetracyclines and erythromycin, acquired resistance is widespread [2]. Due to its activity against many drug-resistant bacteria, vancomycin is one of the most preferred antibiotics in enterococcal infections. However, vancomycin resistance has been increasing in recent years and has been reported as a significant public health issue [8]. Immunosuppressed patients under hospital treatment, such as those being treated for cancer, those receiving organ transplantation, and intensive care patients, are those at the greatest risk for vancomycin-resistant enterococcal (VRE) infections [9]. Linezolid, daptomycin, and tigecycline are last-resort antibiotics in VRE infections. However, resistance to these antibiotics has also been reported [6].

Antimicrobial resistance has become a global concern and a significant public health problem, which has revealed the need for new and effective antimicrobial therapies. Combination antibiotic therapy is a treatment option, especially in nosocomial infections caused by VRE. Because monotherapies are generally bacteriostatic against enterococcal infections and resistance profiles of infecting organisms may not be known, combination antibiotic treatments may be preferred. For example, studies have provided evidence that beta-lactams can increase the effect of daptomycin by changing the VRE membrane potential [10,11]. In considering antibiotic combinations, the impacts of combination may be manifested as indifference, synergy, or antagonism.

One of the necessary needs to combat antimicrobial resistance is the development of new and effective antimicrobial agents. Ceragenins are a new class of antimicrobials designed to meet this need, and their effectiveness has been demonstrated. Ceragenins are non-peptide antimicrobial molecules synthesized from a bile acid and are designed to mimic the morphology and mechanisms of action of endogenous antimicrobial peptides [12]. Many ceragenins were synthesized in the process of structure–activity studies, and ceragenins are numbered sequentially. CSA-13 and CSA-131 are examples of ceragenins that have been studied in detail, and the difference between these compounds is the length of the lipid chain extending from the secondary amine. Longer lipid chains cause ceragenins to cross the outer membrane of Gram-negative bacteria and interact with the cytoplasmic membrane to exert an antibacterial effect. Gram-positive bacteria do not have an outer membrane and are unaffected by the lipid chain. Another difference is that some ceragenins contain an ester structure, while others contain an ether structure. Ester-based compounds (e.g., CSA-44) are easier to prepare on a large scale but are less stable and can spontaneously hydrolyze in water [13]. Prior work has shown that ceragenins are active against drug-resistant Gram-positive bacteria and that these bacteria do not readily become resistant to ceragenins, even after extended exposure. These findings suggest that ceragenins may prove useful in the treatment of infections caused by drug-resistant *Enterococcus* spp. [14]. Therefore, we designed a study to investigate the activities of ceragenins (CSA-13, CSA-44, CSA-90, CSA-131, CSA-138, CSA-142, and CSA-192) and their combinations with linezolid against *Enterococcus* spp.

## 2. Results

### 2.1. Minimum Inhibitory Concentration (MIC) and Minimum Bactericidal Concentration (MBC) Results

The in vitro activities of the ceragenins against 50 Enterococcus species are summarized in Table 1. MIC ranges (μg/mL) for CSA-13, CSA-44, CSA-90, CSA-131, CSA-138, CSA-142, and CSA-192 were 1–32, 1–16, 0.25–8, 0.03–16, 0.03–32, 2–16 and 0.5–32, respectively. The MBC range (μg/mL) for most of the ceragenins was between 1 and 32, with the ranges for CSA-13 and CSA-142 at 8–32 and 4–32, respectively. Since the MIC_50_ and MIC_90_ values of CSA-44 and CSA-192 were the lowest, further studies were conducted with these two ceragenins. According to the findings, the MIC_50_ and MIC_90_ (µg/mL) of linezolid and daptomycin were 0.5/1 and 1/1, respectively. The MICs of linezolid (1 µg/mL) and daptomycin (1 µg/mL) against the quality control strain were within the limits determined by CLSI. Linezolid- or daptomycin-resistant strains were not included in the study.

### 2.2. Checkerboard Results

The results of the combination studies are shown in Table 2. With a fractional inhibitory concentration index (FICI) of ≤0.5 as borderline, synergistic interactions were only seen with the CSA-192-linezolid combination against three *Enterococcus* spp., including one VRE isolate. No antagonism was observed with any combination.

### 2.3. Time Kill Assay Results

**Figure 1:**
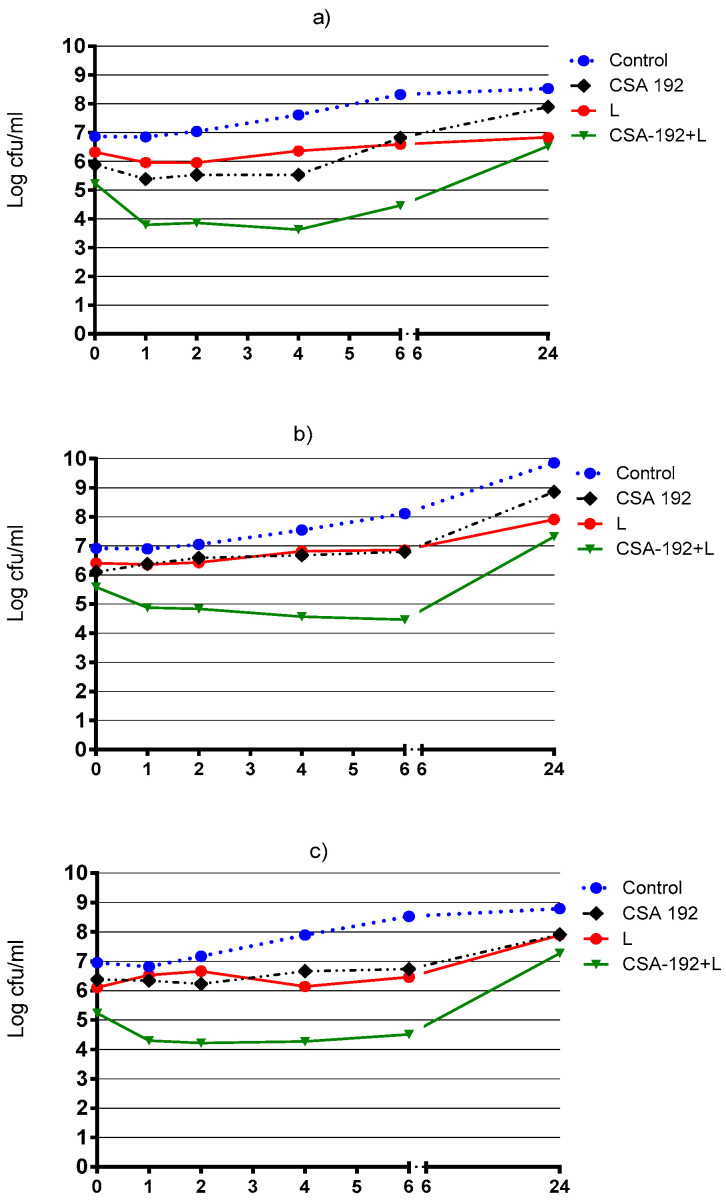
Bactericidal activity of CSA-192 and linezolid (MIC) against three *Enterococcus* spp. strains by using the time-kill curve method. (**a**) VRE strain; (**b**,**c**) vancomycin-susceptible strain L: Linezolid.

Results of time killing curve (TKC) analyses showed rapid bactericidal activity with CSA-192+linezolid within 0 to 6 h for the VRE strain (Figure 1a) and within 6 h for two vancomycin-susceptible *Enterococcus* spp. strains at MIC (Figure 1b,c). The early synergistic interactions of CSA-192+linezolid were also achieved in the first 2–6 h using them in a concentration equal to the MIC.

### 2.4. Effects of CSA-44 and CSA-192 on the Antibacterial Activities of Vancomycin

Effects of CSA-44 and CSA-192 on vancomycin MICs were investigated against a total of 50 *Enterococcus* spp., which 17 were vancomycin-intermediate (*n* = 2) and -resistant strains (*n* = 15). Results are shown in Table 3 (others in Supplementary Materials). MIC changes were seen in the vancomycin+CSA-192 combination with eight strains. The maximum decrease in vancomycin MIC for CSA-44 was 4-fold (with one strain), while for CSA-192, it was up to 32-fold in the MIC (with two strains).

### 2.5. C. elegans Survival Assay

**Figure 2:**
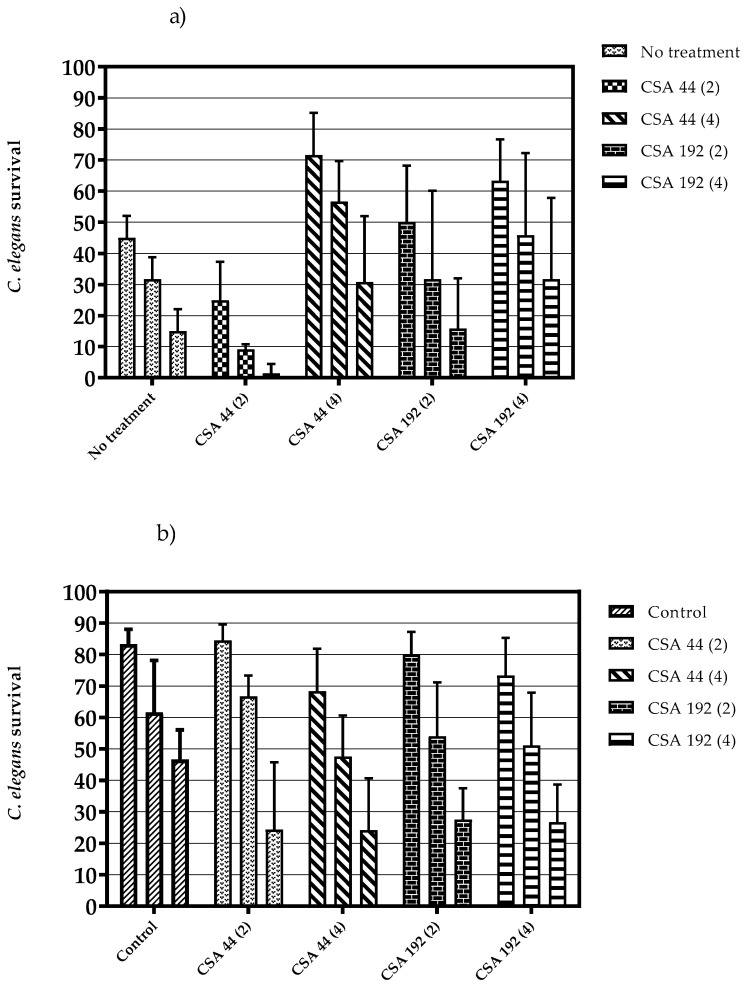
(**a**) Percent survival of infected *C. elegans* (average ± SD) after treatment with CSA-44 and CSA-192 at concentrations of 2 and 4 µg/mL. The results were expressed as the percent survival after 24, 48, and 72 h of infection, respectively. (**b**) Cytotoxicity of ceragenins; nematode survival rates. Compounds were tested twice in each assay. Each assay was replicated at least three times. Statistical analysis for both figures were performed with GraphPad Prism 8. The data were not statistically significant when compared to the control group.

The in vivo effects of CSA-44 and CSA-192 at concentrations 2 and 4 µg/mL were assessed using a *C. elegans* model system. Infection of *C. elegans* with *E. faecalis* ATCC 29212 resulted in decreased nematode survival after 24 h. Increased survival rates were observed with the use of ceragenins compared to no treatment., especially at 4 µg/mL, (Figure 2a). Ceragenins were also non-toxic to *C. elegans*, as measured in comparison to control (Figure 2b).

## 3. Discussion

Antimicrobial resistance is a serious global public health threat with significant health and serious economic consequences. Multidrug-resistant (MDR) pathogens especially constitute a major threat to global human health. This health threat has highlighted the need to identify novel new antimicrobial agents [7,15]. For this purpose, emerging and attractive targets are bacterial membranes due to their preserved structural elements, and generally, cationic amphiphilic molecules such as antimicrobial peptides (AMPs) target bacterial membranes [16]. Ceragenins are designed to mimic the morphology and action of AMPs. They are not peptide-based, are not salt-sensitive, and are relatively simple to prepare and purify on a large scale. Ceragenins cause the formation of transient pores in microbial membranes, resulting in membrane depolarization and cell death, via association with anionic cell surfaces, and they have broad-spectrum antibacterial activity, including drug-resistant strains [17,18].

There are several in vitro and in vivo studies about the successful antimicrobial activities of ceragenins against various Gram-negative and Gram-positive bacteria, some yeast such as *Candida albicans,* and some parasites such as *Trichomonas vaginalis.* These studies showed that CSAs have broad spectrum antimicrobial activities against not only planktonic cells but also the biofilms or spore forms of the microorganisms, and CSA-13 and CSA-131 in particular were found to be the most potent agents [19,20,21,22]. Additionally, a recent study has shown that CSA-44 inhibited biofilm formation and decreased the biofilm mass formed by *E. faecalis* and *C. albicans* on teeth and dental composite surfaces [23]. In this study, we evaluated ceragenins CSA-13, CSA-44, CSA-90, CSA-131, CSA-138, CSA-142, and CSA-192 against clinical *Enterococcus* strains, including VRE isolates. As shown in Table 1, while CSA-90, CSA-131, and CSA-90 showed lower MIC values against some isolates, the lowest MIC_50_ and MIC_90_ results were found with CSA-44 and CSA-192, and we decided to continue a study with them.

Ceragenins not only inhibit bacterial growth, but they also kill bacteria within very similar concentrations (MBC values) as their MIC values. Once the threshold concentration of a ceragenin required for antibacterial activity has been reached, they generally cause cell death, consistent with their mechanism of action [17]. Antibacterial activities of antibiotics are commonly compared using their MIC values, and bactericidal activities are characterized using the MBCs. Similar to our MIC results, the MBC_50_ and MBC_90_ values of evaluated ceragenins were generally equal to or two-fold greater than their MIC values. These results suggested that ceragenins could be used for treating infections in immunocompromised patients who need bactericidal therapy instead of general bacteriostatic monotherapies against *Enterococcus* spp.

Ceragenins display two types of antibacterial activity. The first is a broad-spectrum bactericidal activity against both Gram-negative and Gram-positive organisms. The second is a non-lethal activity that increases bacterial membrane permeability. By increasing the membrane permeability, ceragenins can sensitize bacteria to antibiotics that normally cannot pass through the membranes effectively and do not show strong activity alone [17]. It has been reported by many researchers that, when conventional antibiotics were used together with CSAs, synergistic interactions were observed, and they could provide efficacy at much lower concentrations against microorganisms, including the MDR isolates [20,24,25].

In this study, the effects of CSA-44 and CSA-192 with linezolid or vancomycin were tested against *Enterococcus* spp. Linezolid is an oxazolidinone antibiotic that inhibits protein synthesis by binding to the 50S ribosomal subunit, and it has broad-spectrum Gram-positive activity, including VREs. Nevertheless, like other protein synthesis inhibitors, linezolid is considered a bacteriostatic antibiotic, and its treatment potential for several infections, such as bacteremia or endocarditis, may be limited. We combined CSA-44 or CSA-192 with linezolid using the checkerboard technique, which is the most straightforward and high throughput method available for the assessment of antimicrobial combinations. According to our checkerboard assay, while the CSA-192 and linezolid combinations were generally additive, synergistic interactions were present against 6% of the strains including one VRE. All CSA-44 and linezolid combinations were additive against all 50 *Enterococcus* spp.

The checkerboard technique is a simple and efficient screening test for antibiotic combinations. However, it does not provide any information about the time course of antibiotic activities. This restriction can be overcome using the TKC method. Although TKC experiments are time-consuming and burdensome, they provide a dynamic picture of the efficacy of antibiotics over time. In our study, TKC experiments were performed against three *Enterococcus* strains, one of which was VRE, in which the combination of CSA-192 and linezolid was synergistic, thus confirming the results obtained via the checkerboard method. According to these results, it was observed that linezolid and CSA-192, at MIC values, showed very close curves to each other and had bacteriostatic effects, but no bactericidal activity (≥3 log reduction) was observed. Synergism was found in the first 2–6 h for all three isolates. Similarly, Chin et al. [26] demonstrated that there was no difference in the log killing at 24 h with CSA-13 in combination with linezolid or vancomycin,

Vancomycin is a glycopeptide antibiotic that inhibits cell wall synthesis and is regarded as a medication of last resort for treating life-threatening infections caused by Gram-positive bacteria. The evolution of bacterial resistance to vancomycin is a growing problem, especially within healthcare facilities such as hospitals. The widespread use of vancomycin makes drug resistance a significant concern. In this study, due to the high vancomycin MIC values of VRE isolates over the toxic therapeutic doses, we decided to test the potential reduction in MICs by adding ceragenins at a constant concentration rather than using the checkerboard technique. When we tested the antibiotic adjuvant effects of CSA-44 or CSA-192 against vancomycin MIC values in VRE isolates, we observed decreased MIC values up to 32-fold with CSA-192 with eight isolates. In comparison, CSA-44 decreased the MICs between 2- and 4-fold in three isolates. Considering that at least a 4-fold decrease in MIC values can be considered synergism, it can be concluded that the combination of CSA-192 and vancomycin is synergistic against eight VRE isolates. These results suggested that the combined effects of inhibition of cell wall synthesis and perturbation of membrane potential inhibit bacterial growth.

Although in vitro studies with antimicrobial agents are important and may provide data for characterizing antibiotics, they can only be applied in clinics if confirmed by the results obtained in in vivo experiments. At the beginning of infection research around the late 19th century, animal infection experiments were intended to identify causative pathogens and toxins. Recently, animal models have generally been used to determine the impacts of new molecules against infectious processes and find new targets for anti-infective drugs. To perform infection experiments using mammals such as mice and rabbits, research requires large numbers of animals and must adhere to international ethical guidelines. *C. elegans*, an important model organism in embryology, began to be utilized as an animal infection model of *Pseudomonas aeruginosa* in the late 1990s [27]. *C. elegans* is normally fed with *Escherichia coli*, but the nematode dies when it is fed with other, more pathogenic bacteria. *C. elegans* is an attractive infection modeling method due to the small body size, easy and low-cost production in the laboratory, and not being subject to the ethical procedures required in animal models [27,28]. This study evaluated CSA-44 and CSA-192 using the *C. elegans* infection model at their MIC_50_ and MIC_90_ values, 2 and 4 µg/mL, respectively. When treated with these ceragenins after being infected with *E. faecalis*, the survival rates of the *C. elegans* were increased comparing the untreated control, especially with MIC_90_ values of ceragenins. Also, we tested the ceragenins for toxic side effects of these ceragenins with *C. elegans*, and we found that they were non-toxic for *C. elegans*.

In conclusion, we evaluated a group of lead ceragenins against the *Enterococcus* spp., which are leading causes of healthcare-associated infections worldwide, in particular urinary tract, soft tissue, and device-related infections. These ceragenins were found to be effective with similar MIC values, regardless of the antibiotic resistance status of the bacteria. Among these, CSA-44 and CSA-192 were the most active agents according to their MIC_50_ and MIC_90_ values at 2 and 4 µg/mL, respectively. MBC values, which gave the bactericidal activities of ceragenins, were also independent of the antibiotic resistance of the bacteria and were generally found at concentrations equal to or twice the MIC values. When we combined the ceragenins with conventional antibiotics such as linezolid, the combinations had a generally additive effect, and the synergism was observed for 6% of isolates with CSA-192. The TKC studies confirmed the early synergistic interactions between CSA-192 and linezolid in 2–6 h. We also tested these ceragenins with vancomycin at a constant concentration and found that CSA-192 significantly reduced vancomycin MICs up to 32 times and CSA-44 up to 4 times. Finally, the effects of these ceragenins in an *E. faecalis* in vivo infection model with *C. elegans* were evaluated, and significant increases in the number of surviving nematodes with CSA-44 and CSA-192 were observed without any cytotoxic effects.

## 4. Materials and Methods

### 4.1. Strains and Culture Conditions

A total of 50 non-repeated *Enterococcus* spp. (17 of them VRE) isolated from various clinical specimens including blood (*n* = 10), urine (*n* = 22), vaginal fluid (*n* = 17), and prostatic fluid (*n* = 1) were submitted to the Synevo Laboratories Ankara Central Laboratory in Turkey (2021–2022). Before analysis, each isolate was cultured on tryptic soy agar (Difco Laboratories, Detroit, MI, USA) plates to ensure viability. *E. faecalis* ATCC 29212 was used as a quality control strain. *Caenorhabditis elegans* N2 (glp-4; sek-1) was propagated using standard conditions, synchronized via hypochlorite bleaching, and cultured on nematode growth medium using *Escherichia coli* OP50 as a food source [29,30].

### 4.2. Antimicrobial Agents

**Figure 3:**
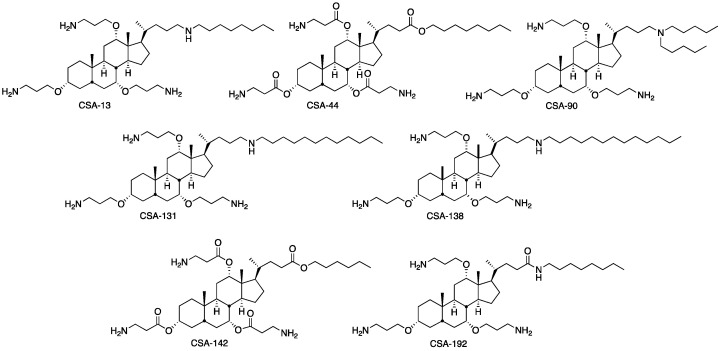
Chemical structures of studied CSAs.

CSA-13, CSA-44, CSA-90, CSA-131, CSA-138, CSA-142, and CSA-192 were synthesized from a cholic acid scaffold method as described previously [31,32] (Figure 3). Antibiotics were kindly provided by their manufacturers such as linezolid and vancomycin from Koçak Farma İlaç ve Kimya Sanayi A.Ş., and daptomycin from Novartis Sağlık, Gıda, Tarım Ürünleri San. Tic. A.Ş. Stock solutions of ceragenins and antibiotics were prepared from dry powders in distilled water. Frozen solutions of antimicrobial agents were used within six months.

### 4.3. Determination of MIC and MBC

The antimicrobial activities of ceragenins and antibiotics against *Enterococcus* strains were determined by using the microbroth dilution technique according to the Clinical and Laboratory Standards Institute [33]. The inoculum was prepared using overnight cultures of the bacteria in a final concentration of 1 × 10^6^ colony forming units/mL. Double dilutions of antimicrobials, with a range of concentrations of 128–0.015 µg/mL were tested in antimicrobial activity studies. Plates were incubated at 37 °C 24 h. The MIC was defined as the lowest antimicrobial concentration that completely inhibited the visible growth of microorganisms. Moreover, MBCs of ceragenins were determined by subculturing 10 µL samples from each well that demonstrated no visible growth by plating on TSA. After 24 h incubation at 37 °C, resultant colonies were counted, and the MBC was defined as the lowest antimicrobial concentration that produced at least a 99.9% decrease in microorganism counts [34]. Determination of MIC_50_ and MIC_90_ involved calculating the lowest concentration of the antimicrobial capable of inhibiting 50% and 90% of the bacterial isolates, respectively. MBC_50_ and MBC_90_ were calculated based on the same formula.

### 4.4. Determination of FICI

Interactions between the CSA-44 or CSA-192, which present the lowest MIC_50_ and MIC_90_ results via the microdilution method, and linezolid were tested against *Enterococcus* strains, using the microbroth checkerboard technique [35]. Each microtiter well, containing a mixture of CSA-44 or CSA-192 and linezolid in different final concentrations ranging from 2xMIC to 1/8xMIC, was inoculated with fresh culture. After an 18–20 h incubation period at 37 °C, the FIC index was calculated using the following formulas: FICA = (MICA in combination)/(MICA alone), FICB = (MICB in combination)/(MICB alone), and the FIC index = FICA + FICB. The combination value was determined from the highest dilution of the antimicrobial combination that permitted no visible growth. With this method, a FICI of ≤0.5 was considered synergistic, of >0.5–4 was considered to be additive, and of >4.0 was considered to be antagonistic [36]. The experiments were generally carried out in triplicate.

### 4.5. Time Kill Assays

The TKC method was monitored to observe the dynamic killing kinetics of the CSA-44 or CSA-192 and linezolid combinations, which were significantly synergistic in checkerboard assays [34]. The TKCs were constructed by plotting the mean colony counts (log cfu/mL) of CSA-44 or CSA-192 and linezolid alone and in combination with versus time. The bacterial suspensions were incubated with antimicrobials or their combinations at 37 °C with gentle shaking. The viable bacterial counts were determined after 0, 1, 2, 4, 6, and 24 h of incubation. One milliliter of the suspension was taken and diluted sequentially with sterile saline solution. Next, 100 µL of the bacterial suspensions or dilutions were applied to the TSA plates and left to incubate overnight at 37 °C to determine the cfu. For each strain, an antimicrobial-free control was included. The lowest detection limit for the time kill assays was 1 log_10_ cfu/mL. The antimicrobial carry-over was controlled by inhibiting the colonial growth at the side of the initial streak according to the guidelines. The effects of the combination were analyzed in comparison with those of the most active agent alone. Synergy was defined as a 2 log cfu/mL decrease, in the colony counts at 24 h. The definition of bactericidal activity was based on a decrease of ≥3 log cfu/mL from the initial inoculum [34].

### 4.6. MICs of Vancomycin in the Presence of Ceragenins

The effects of CSA-44 and CSA-192 on the MIC values of vancomycin against both vancomycin-resistant and vancomycin-susceptible *Enterococcus* spp. were investigated. For this purpose, serial twofold dilutions of vancomycin ranging from 512 to 1 μg/mL were prepared in Mueller-Hinton broth (MHB) (Difco, Detroit, MI, USA), and then ceragenins were added to microplate wells (same concentration in all wells for each bacterium according to MIC values). The MICs of vancomycin in the presence of ceragenins were defined as the lowest antimicrobial concentrations that completely inhibited the visible growth of microorganisms.

### 4.7. Caenorhabditis elegans Survival Assay

For the *C*. *elegans* viability test, synchronized worms (L4 stage) were suspended in OGM medium (95% M9 buffer, 5% brain heart infusion broth, 10 μg/mL cholesterol) and added into 96-well plates with a minimum of 20 worms per well [27,28]. Nematodes were infected with 25 μL of overnight culture of *E. faecalis* ATCC 29212 adjusted to 2 × 10^9^ cfu/mL in OGM medium and exposed to 25 μL of treatment with two different concentrations of CSA-44 and CSA-192 alone. Infected but untreated nematodes were defined as “No treatment”. Cytotoxicity wells containing only ceragenins and nematodes were incorporated into the experiments. Uninfected nematodes were employed as control groups in cytotoxicity assays conducted in the OGM medium.

The assay plates were incubated at 25 °C for up to 3 days and the number of viable and dead nematodes was assessed every 24 h. The fraction of dead worms was determined by counting the number of dead worms and the total number of worms in each well.

### 4.8. Statistical Analysis

Statistical analysis of the data was carried out using GraphPad Prism 8 (GraphPad Software, La Jolla, CA, USA). Data for *C. elegans* survival assay underwent a two-way ANOVA analysis followed by Dunnett’s test to determine statistical significance. A *p*-value of < 0.05 was considered statistically significant.

## Figures and Tables

**Table 1:** The MIC and MBC values of ceragenins against *Enterococcus* spp. (µg/mL).

		MIC Range	MIC_50_	MIC_90_	MBC Range	MBC_50_	MBC_90_
**CSA-13**	VSE	2–32	16	16	4–32	16	32
	VRE	1–16	8	16	2–32	8	32
**CSA-44**	VSE	1–16	2	4	1–32	4	32
	VRE	1–4	2	4	1–16	4	4
**CSA-90**	VSE	1–8	8	8	1–32	8	32
	VRE	0.25–8	2	4	0.5–16	4	16
**CSA-131**	VSE	0.5–16	16	16	1–32	16	32
	VRE	0.03–16	16	16	1–32	16	32
**CSA-138**	VSE	1–32	16	16	1–32	16	32
	VRE	0.03–16	8	16	1–32	16	32
**CSA-142**	VSE	2–16	16	16	4–32	16	32
	VRE	4–16	8	16	8–32	16	32
**CSA-192**	VSE	0.5–32	2	4	1–32	4	32
	VRE	0.5–4	1	2	1–8	4	8

VSE: Vancomycin-susceptible *Enterococcus* spp. VRE: Vancomycin-resistant *Enterococcus* spp. MIC_50_: MIC that inhibited 50% of the tested microorganisms. MIC_90_: MIC that inhibited 90% of the tested microorganisms. MBC_50_: MBC that killed 50% of the tested microorganisms. MBC_90_: MBC that killed 90% of the tested microorganisms.

**Table 2:** The effects of CSA-44 or CSA-192 and linezolid combinations. Numbers of strains with the indicated characteristics are given.

	FIC Index		
	≤0.5 (Synergistic)	>0.5–4 (Additive)	>4.0 (Antagonistic)
**CSA-44+Linezolid**	-	50	-
**CSA-192+Linezolid**	3	47	-

**Table 3:** The effects of CSA-44 and CSA-192 on the antibacterial activities of vancomycin.

MIC Values (µg/mL)					
Isolates	CSA-44 Alone	CSA-192 Alone	Vancomycin Alone	Vancomycin +CSA-44	Vancomycin +CSA-192
**E1**	2	2	8	8	8
**E2**	2	1	16	4	4
**E3**	2	2	32	16	8
**E4**	1	1	32	32	1
**E5**	1	1	32	16	4
**E6**	1	1	128	128	128
**E7**	1	1	128	128	128
**E8**	1	1	256	256	16
**E9**	1	1	256	256	256
**E10**	4	0.5	256	256	64
**E11**	1	1	512	512	512
**E12**	4	2	512	256	32
**E13**	2	4	512	512	512
**E14**	2	2	512	512	512
**E15**	2	2	512	512	512
**E16**	2	2	512	512	128
**E17**	2	2	512	512	512

## Data Availability

Data is contained within the article and supplementary material.

## Supplementary Materials

The following supporting information can be downloaded at: https://www.mdpi.com/article/10.3390/ph16121643/s1, Table S1: The effects of CSA-44 and CSA-192 against antibacterial activities of vancomycin (Vancomycin-susceptible strains).
