# Supplementary material for: Antimicrobial Activity of Ceragenins against Vancomycin-Susceptible and -Resistant Enterococcus spp
Source: Pharmaceuticals (Basel). 2023 Nov 23;16(12):1643. doi: 10.3390/ph16121643 (PMC10747769; doi:10.3390/ph16121643)
Supplement: Supplementary file 1 [file pharmaceuticals-16-01643-s001.zip › pharmaceuticals-2573443-supplementary.pdf]

Supplement material Table S1: The effects of CSA-44 and CSA-192 against antibacterial activities of vancomycin (Vancomycin susceptible strains)

	MIC values (µg/ml)		
**Isolates**	**Vancomycin alone**	**Vancomycin +CSA-44**	**Vancomycin +CSA-192**
**1**	0.5	0.5	0.5
**2**	0.5	0.5	0.5
**3**	1	1	0.5
**4**	0.5	0.5	0.5
**5**	2	0.5	0.5
**6**	2	2	0.5
**7**	1	0.5	0.5
**8**	0.5	0.5	0.5
**9**	2	1	1
**10**	1	0.5	1
**11**	2	0.5	0.5
**12**	0.5	0.5	0.5
**13**	0.5	0.5	0.5
**14**	1	1	1
**15**	2	0.5	0.5
**16**	0.5	0.5	1
**17**	1	1	1
**18**	1	0.5	0.5
**19**	0.5	0.5	0.5
**20**	0.5	0.5	0.5
**21**	0.5	0.5	0.5
**22**	1	0.5	1
**23**	1	0.5	1
**24**	2	0.5	0.5
**25**	1	0.5	1
**26**	0.5	0.5	0.5
**27**	0.5	0.5	0.5
**28**	0.5	0.5	0.5
**29**	2	0.5	0.5
**30**	0.5	0.5	0.5
**31**	0.5	0.5	0.5
**32**	1	1	0.5
**33**	1	1	0.5
